# Europe, different approaches to similar problems

**DOI:** 10.1186/1472-6955-14-S1-K2

**Published:** 2015-10-08

**Authors:** Enric Mayolas

**Affiliations:** 1 ICU specialist, Hospital & Health Care Consultant, Spain

## 

Europe is a political reality, 28 countries with health systems with full responsibility of each member state, without a common portfolio of services and structures for planning, management and control. In terms of servicing two major agreements: emergency care through UE and more recently Cross-border Health Care Directive implemented October 2013. However the cover (portfolio of services), the provision, professional contracts, planning, financing are different and always taking into account the limits of national borders that no longer exist.

The analysis of indicators of resources and health show relations according to the financing and payment systems, and less relation to the level of spending.

The services and emigration policies show the difference between countries and the difficulty to have a common European policy to access health services.

The challenges facing individual countries are common, although the solutions adopted are very different. Proposals made of the Future Hospital Commission (Royal College of Physicians UK) analysing their health care situation, is very common throughout the EU, and measures proposed could be largely adopted.

